# Mechanical pain sensitivity of deep tissues in children - possible development of myofascial trigger points in children

**DOI:** 10.1186/1471-2474-13-13

**Published:** 2012-02-08

**Authors:** Ting-I Han, Chang-Zern Hong, Fang-Chuan Kuo, Yueh-Ling Hsieh, Li-Wei Chou, Mu-Jung Kao

**Affiliations:** 1 Department of Physical Medicine and Rehabilitation, China Medical University Hospital, Taichung, Taiwan; 2 Department of Physical Therapy, Hungkuang University, Taichung, Taiwan; 3 Department of Physical Therapy and Graduate Institute of Rehabilitation Science, China Medical University, Taichung, Taiwan; 4 School of Chinese Medicine, College of Chinese Medicine, China Medical University, Taichung, Taiwan; 5 Department of Physical Medicine and Rehabilitation, Taipei City Hospital, Taipei, Taiwan; 6 Department of Physical Therapy and Assistive Technology, National Yang-Ming University, Taipei, Taiwan

**Keywords:** myofascial trigger points, children, pressure pain threshold, algometry

## Abstract

**Background:**

It is still unclear when latent myofascial trigger points (MTrPs) develop during early life. This study is designed to investigate the mechanical pain sensitivity of deep tissues in children in order to see the possible timing of the development of latent MTrPs and attachment trigger points (A-TrPs) in school children.

**Methods:**

Five hundreds and five healthy school children (age 4- 11 years) were investigated. A pressure algometer was used to measure the pressure pain threshold (PPT) at three different sites in the brachioradialis muscle: the lateral epicondyle at elbow (site A, assumed to be the A-TrP site), the mid-point of the muscle belly (site B, assumed to be the MTrP site), and the muscle-tendon junction as a control site (site C).

**Results:**

The results showed that, for all children in this study, the mean PPT values was significantly lower (*p *< 0.05) at the assumed A-TrP site (site A) than at the other two sites, and was significantly lower (*p *< 0.05) at the assumed MTrP site (site B) than at the control site (site C). These findings are consistent if the data is analyzed for different genders, different dominant sides, and different activity levels.

**Conclusions:**

It is concluded that a child had increased sensitivity at the tendon attachment site and the muscle belly (endplate zone) after age of 4 years. Therefore, it is likely that a child may develop an A-Trp and a latent MTrP at the brachioradialis muscle after the age of 4 years. The changes in sensitivity, or the development for these trigger points, may not be related to the activity level of children aged 7-11 years. Further investigation is still required to indentify the exact timing of the initial occurrence of a-Trps and latent MTrPs.

## Background

Myofascial trigger points (MTrPs) are a major cause of muscle pain in clinical practice. MTrPs are defined as a hyperirritable area in a taut band of skeletal muscle fibers that experience referred pain and local twitch responses. An active MTrP is painful either spontaneously or during movement, whereas a latent MTrP is painful only in response to pressure or compression (tenderness). MTrPs are characterized by an exquisite tender spot, a taut band, referred pain, a local twitch response, motor dysfunction, and an autonomic phenomenon [1]. Latent MTrPs can reportedly be activated by a lesion at other sites or within the muscle itself via central sensitization, whereas active MTrPs can be permanently inactivated completely only if the underlying etiologies have been eliminated [1-8]. According to clinical observation, myofascial pain can be suppressed by effective myofascial pain therapy, such as MTrP injections; however, the pain often recurs after a few days or a few weeks if the related pathological lesion is not eliminated [2,5]. Persistent or recurrent MTrPs are usually related to remote lesions. It has been reported that the number and pain intensity of MTrPs were significantly reduced after treatment of lumbar disc herniation [9]. The association of active MTrPs with cervical disc lesions [10], cervical facet lesions [11], cervical radiculopathy, lumbar disc lesions, osteoarthritis of the knee [12], teres minor tendinitis [13], lateral epicondylitis, floating kidney [14], septic arthritis [15], or herpes zoster [16] has been reported in previous studies. Cervical spine manipulation [17] or local facet joint injection [18] could effectively relieve the pain from MTrPs. Activation of MTrPs can cause pain; any movement that could interfere with the healing process of the primary lesion should be avoided. Muscle pain can be an important defense mechanism for avoiding further injury before complete healing of the etiological lesion [4,7]. However, the mechanism for the development of latent MTrP is still uncertain. Latent MTrPs can affect almost all "normal" (non-painful) muscles in normal adults. No distinct MTrP has been identified in infants less than one year old [19]. However, the mechanism underlying the development of latent MTrP during early life remains unknown.

Fischer further defined an attachment trigger point (A-Trp) as a point at the bony attachment (enthesis) of a muscle or tendon [1,20,21], located at the end of a taut band. Compression of an A-TrP of a certain muscle can elicit pain locally and referred pain in the central MTrP of this muscle [21]. Sometimes, a local twitch response can also be elicited by the compression of an A-TrP. A-TrPs can commonly be found in the attachment site of a frequently used muscle, such as the brachioradialis or extensor carpi radialis of adults.

After comprehensive studies on both human subjects and rabbits [1,3,6-8], Hong has defined an MTrP as the "accumulation of sensitized nociceptors in the endplate zone of muscle fibers, and the irritability of MTrP is proportionate to the amount of sensitized nociceptors [6,8]. The key point of an MTrP is the amount of sensitized nociceptors, rather than the associated phenomena (pain, referred pain, local twitch response, motor dysfunction, autonomic phenomena, etc). Therefore, one can consider a hyperirritable spot as an MTrP either referred pain, local twitch response, or autonomic phenomena can be identified or not, since the associated phenomena may or may not be identified, depending on the irritability of this MTrP. The irritability can be measured as degree of pain and referred pain, prevalence of local twitch responses [22] and endplate noise [23]. A pressure algometer was designed for the measurement of MTrP irritability based on pressure pain threshold (PPT) values [24]. This algometer has been considered as a reliable and valid way of measuring MTrP sensitivity in previous studies [25-28]. MTrP irritability assessed with subject pain intensity is proportionate to the PPT value measured on the MTrP [23,29].

Using a pressure algometer, the current study investigates the mechanical pain sensitivity of deep tissues in children in order to see the possible timing of the development of latent MTrPs and A-TrPs based on the irritability assessment of these points in schoolchildren across different age groups.

## Methods

To investigate the possible development of A-TrPs and MTrPs, the irritability at three different sites (sites A, B, and C, at the assumed A-TrP, MTrP, and muscle-tendon junction site) of bilateral brachioradialis muscles of children 4-11 years old were measured with a pressure algometer and compared in terms of PPT values.

A total of 505 healthy schoolchildren (258 males and 247 females) were recruited from 15 different schools (9 kindergartens and 6 elementary schools), with the consent of schoolteachers, the participating children, and their legal guardians. The study was approved by a qualified institutional review board. All children or their legal guardians signed informed consent forms. The exclusion criteria for the subjects included any acute or serious illness, any recent trauma to the upper limbs, any upper limb deformities, any communication problems, and any emotional instability. Table 1 shows the demographic data of all subjects, including gender, the dominant side of the subjects (handedness), and age distribution. The mean age of the subjects was 7.9 ± 2.1 years, without significant differences in the distribution or mean ages between genders.

**Table 1:** Demographic Data of Subjects

	All subjects	Boys	Girls	♂ *vs *♀^a^
Total number of Children	505	258	247	
Age (years)	7.9 ± 2.1	7.8 ± 2.1	8.0 ± 2.1	P > .05
(Range)	(4-11)	(4-11)	(4-11)	
Dominant side				
Right	480	240	240	P > .05
Left	25	18	7	
Children attended extra-sports	80	51	29	P > .05
Age group:				P > .05
4 years	33	17	16	
5 years	59	35	24	
6 years	45	27	18	
7 years	71	33	38	
8 years	75	36	39	
9 years	88	47	41	
10 years	77	39	38	
11 years	57	24	33	

^a ^tested with Student's t-test.

A total of 80 children 7 years old and older (51 boys and 29 girls) participated in regular exercise programs after school, including track and field events, swimming programs, karate practice, skating practice, basketball training courses, table tennis training courses, badminton training courses, and dancing courses. These subjects were considered as the "active children." The rest of the children attended regular physical education courses at school. Among them, 288 children (128 boys and 160 girls) 7 years old and older were considered "normal children" for comparison with the "active children" to investigate the influence of activity level.

The brachioradialis muscle originates from the lateral supracondylar ridge of the humerus and the lateral intermuscular septum distal to where the radial nerve penetrates the septum at mid-arm level, and inserts into the styloid process of the radius. The endplate zone, the assumed site of the MTrP, is in the middle portion of the muscle [1]. This muscle is easily identified when the subject attempts to flex the elbow against resistance at 90 degrees of flexion.

**Figure 1:**
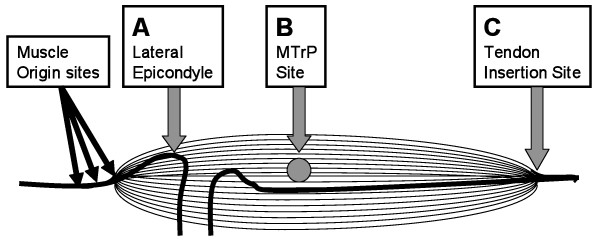
**Sites of measurement**.

The same measurement methods were followed for the identification of the three sites on the brachioradialis muscle as previously described [19]. At first, the distance between the lateral epicondyle and the styloid process was measured. The midpoint of this distance was considered as the junction of the muscle and tendon. The assumed MTrP (site B) was approximately placed proximally at a point one-third the distance between this junction site and the lateral epicondyle [1]. It is located at the most prominent portion of the muscle belly (endplate zone). The assumed A-TrP (site A) was the most proximal portion of the lateral epicondyle, i.e., the region just above the original site of hand/finger extensors, because the exact origin of the brachioradialis is difficult to identify. As shown in Figure 1, these three sites were measured for pressure pain threshold using a pressure algometer: Site A is the assumed A-TrP site, Site B is the assumed MTrP site, and Site C, the muscle-tendon junction site, acted as the control site.

The pressure algometer was placed on the corresponding site to be measured [24]. First, the procedures were explained clearly to the child. The child was in the sitting position, made comfortable, and encouraged to remain completely relaxed. The round rubber end of the algometer was in full contact with the skin and the force transmission rod was perpendicular to skin surface. The compression pressure was increased gradually at a rate of approximately 1 kg/sec. The child was asked to say "YES" when he/she begins to feel pain or discomfort, at which point, the examiner stopped the compression and read the scale on the algometer (kg/cm^2^), indicating the PPT value at that site. The child was asked to remember this level of pain discomfort and to apply the same criterion for the next measurement. The subjects might demonstrate pain responses such as pulling away, grimacing, crying, indicating that the pain threshold had been exceeded [20,24]. If this was the case, the subject was given instructions again, and a repeat measurement was taken to ensure the "real" threshold is obtained. Three measurements were performed at each site, and a total of nine measurements were performed for the three sites within each muscle. At least one minute of rest was allowed between consecutive measurements. The sequence of measurement at the three sites was randomly assigned. Only one well-trained examiner conducted the measurements throughout the whole course of this study.

### Statistical analysis

The mean PPT values obtained from the three repeated measures at each site were collected for data analysis. For each child, the mean PPT values from six recording sites (three sites on each side) were collected. The differences in PPT among the three sites were analyzed using a one-way repeated measures ANOVA. Gender differences, handedness differences, and the differences between the active group and the normal group were analyzed by t-test. For analyses of PPT differences between the "active" and "normal" group, the "percentage differences between site A or B and site C" was defined as [(PPT value at site C) - (PPT value at site A or B)]/(PPT value at site C) for comparison. A *p*-value of less than 0.05 was considered statistically significant.

## Results

**Figure 2:**
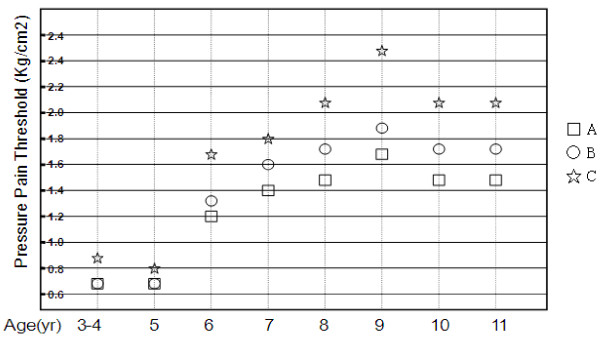
**Changes in pressure pain threshold at different points of brachioradialis in children when growing up**.

For all children, when both right and left sides were combined, the site A (assumed A-TrP) had the lowest (*p *< 0.05) mean PPT value, whereas the site C (muscle-tendon junction site) had the highest (*p *< 0.05) value (Table 2). The mean PPT value was significantly lower at site A than site B (assumed MTrP site) or site C (*p *< 0.05). Furthermore, it was significantly lower (*p *< 0.05) at site B than at site C. As shown in Figure 2, the mean PPT values at any measured site tend to increase with age up to 9 years old, and then decreased slightly beyond 10 years old.

**Table 2:** Pressure Pain Threshold (kg/cm^2^) at 3 Sites (A, B, and C) of Brachioradialis Muscles (combined right and left sides) in Different Age Groups

Measured Sites	A	B	C	A *vs *B ^a^	A *vs *C ^a ^*	B *vs *C ^a^
All Children (n = 1,010)	1.36 ± 0.63	1.52 ± 0.75	1.88 ± 0.98	P < .05	P < .05	P < .05

4 years-old (n = 66)	0.66 ± 0.29	0.74 ± 0.38	0.85 ± 0.49	P < .05	P < .05	P < .05

5 years-old (n = 118)	0.65 ± 0.31	0.71 ± 0.37	0.80 ± 0.47	P < .05	P < .05	P < .05

6 years-old (n = 90)	1.20 ± 0.49	1.35 ± 0.57	1.67 ± 0.79	P < .05	P < .05	P < .05

7 years-old (n = 142)	1.42 ± 0.39	1.59 ± 0.60	1.85 ± 0.63	P < .05	P < .05	P < .05

8 years-old (n = 150)	1.49 ± 0.50	1.67 ± 0.56	2.12 ± 0.79	P < .05	P < .05	P < .05

9 years-old (n = 176)	1.73 ± 0.82	1.94 ± 0.99	2.50 ± 1.29	P < .05	P < .05	P < .05

10 years-old (n = 154)	1.54 ± 0.53	1.74 ± 0.60	2.14 ± 0.76	P < .05	P < .05	P < .05

11 years-old (n = 114)	1.55 ± 0.37	1.73 ± 0.43	2.14 ± 0.64	P < .05	P < .05	P < .05

Measured site A = Attachment trigger point; Measured site B = Myofascial trigger point; Measured site C = muscle-tendon junction; ^a ^tested with one way repeated measure ANOVA

For each measured site, no significant difference (*p *> 0.05) in mean PPT values was observed between boys and girls for all different age groups (Table 3). When boys or girls were considered separately, the mean PPT values were always significantly (*p *< 0.05) lower at either site A or site B than that at site C, and were also significantly lower at site A than at site B (Table 3).

**Table 3:** Pressure Pain Threshold (kg/cm^2^) at 3 Sites (A, B, and C) of Brachioradialis Muscles (combined right and left sides) in Different Sex

Measured Sites	A	B	C	A *vs *B ^a^	A *vs *C ^a^	B *vs *C ^a^
All Children (n = 1,010)	1.36 ± 0.63	1.52 ± 0.75	1.88 ± 0.98	P < .05	P < .05	P < .05

Boys (n = 516)	1.35 ± 0.61	1.51 ± 0.70	1.859 ± 0.93	P < .05	P < .05	P < .05

Girls (n = 494)	1.37 ± 0.65	1.53 ± 0.79	1.90 ± 0.980	P < .05	P < .05	P < .05

Boys *vs* Girls	P > .05	P > .05	P > .05			

Measured site A = Attachment trigger point; Measured site B = Myofascial trigger point; Measured site C = muscle-tendon junction; ^a ^tested with one way repeated measure ANOVA.

No significant difference (*p *> 0.05) was observed between the dominant and non-dominant sides for each measured site (Table 4). For both the dominant and the non-dominant side, the mean PPT values were significantly (*p *< 0.05) lowest at site A, followed by site B and highest at site C (Table 4).

**Table 4:** Pressure Pain Threshold (kg/cm^2^) at 3 Sites (A, B, and C) of Brachioradialis Muscles in Dominant and Non-dominant Sides

Measured Sites	A	B	C	A *vs *B ^a^	A *vs *C ^a^	B *vs *C ^a^
Both sides (n = 1,010)	1.36 ± 0.63	1.52 ± 0.75	1.88 ± 0.98	P < .05	P < .05	P < .05

Dominant side (n = 505)	1.32 ± 0.60	1.47 ± 0.70	1.84 ± 0.96	P < .05	P < .05	P < .05

Non-dominant side (n = 505)	1.40 ± 0.67	1.58 ± 0.82	1.91 ± 1.02	P < .05	P < .05	P < .05

Dominant *vs* Non-dominant	P > .05	P > .05	P > .05			

Measured site A = Attachment trigger point; Measured site B = Myofascial trigger point; Measured site C = muscle-tendon junction; ^a ^tested with one way repeated measure ANOVA.

The influence of activity level on PPT value was analyzed only for children 7 years old and older based on the percentage difference in PPT values between the site C and site A or site B (Table 5). The PPT values for site A and site B were not significantly different between the "active" and the "normal" children, both when assessed overall and when each age group was assessed independently (Table 5).

**Table 5:** Pressure Pain Threshold (kg/cm^2^) at 3 Sites (A, B, and C) of Brachioradialis Muscles (combined right and left sides) in Children with Different Activity Levels

	Active Children	Normal children	P values ^a^
All Children - age 7 -11 (n = 736)	n = 160	n = 576	
% PPT difference at A	27.5 ± 9.8	25.7 ± 11.9	P > .05
% PPT difference at B	17.7 ± 8.4	17.8+13.8	P > .05

7 years-old (n = 142)	n = 4	n = 138	
% PPT difference at A	33.4 ± 9.5	19.5 ± 13.2	P > .05
% PPT difference at B	39.2 ± 25.4	23.4 ± 50.4	P > .05

8 years-old (n = 150)	n = 20	n = 130	
% PPT difference at A	30.4 ± 9.8	27.5 ± 11.5	P > .05
% PPT difference at B	46.8 ± 25.2	45.4 ± 37.1	P > .05

9 years-old (n = 176)	n = 40	n = 136	
% PPT difference at A	28.9 ± 11.3	28.9 ± 10.7	P > .05
% PPT difference at B	50.0 ± 49.4	57.0 ± 42.5	P > .05

10 years-old (n = 154)	n = 52	n = 102	
% PPT difference at A	26.3 ± 7.4	27.3 ± 9.6	P > .05
% PPT difference at B	36.8 ± 24.6	40.7 ± 27.0	P > .05

11 years-old (n = 114)	n = 44	n = 70	
% PPT difference at A	26.0 ± 10.6	25.7 ± 11.2	P > .05
% PPT difference at B	36.4 ± 21.6	43.9 ± 33.1	P > .05

Active children = children doing additional regular sports activity after school.

Normal children = children doing no additional sports activity after school.

PPT = pressure pain threshold (kg/cm^2^).

% PPT difference at A = [(PPT at C) - (PPT at A)]/(PPT at C) × 100%

% PPT difference at B = [(PPT at C) - (PPT at B)]/(PPT at C) × 100%

^a ^tested with Student's t-test.

## Discussion

In the current study, the origin (assumed A-TrP site) of the brachioradialis muscle was more irritable than the middle of the muscle belly (endplate zone, assumed MTrP site), and the muscle-tendon junction of the control site. This occurred in children of both genders 4 years and older. Therefore, we have assumed that children may develop latent MTrP at the belly of the brachioradialis muscle at 4 years old. However, the exact timing of the development of the latent MTrP is still unclear and requires further investigation. The timing of development of a latent MTrP may differ from muscle to muscle.

In a previous similar study on the brachioradialis muscles of 60 newborns comparing to 60 healthy adults, Kao et al [19] found no significant differences in mean PPT values measured at site A, site B, and site C in infants, but the mean PPT values at site B was significantly lower than that in site A or site C.

The irritability of all sites in the brachioradialis muscle tended to decrease gradually with age until 9 years old. Thus, children younger than 5 years old have a very low pain threshold (Table 2), but this pain threshold gradually increases as they grow up. The pain threshold reaches a plateau at 9 years old. However, the pain threshold (mean PPT) at the endplate zone of muscle belly (assumed MTrP region) of the brachioradialis muscle in children aged 9-11 years is still much lower than that in adults [19]. In an earlier study on the same muscle, brachioradialis, Kao et al found that the mean PPT values at the site B (assumed MTrP region) was 2.54 ± 0.87 kg/cm^2 ^in the right side and 2.87 ± 0.52 kg/cm^2 ^in the left side of brachial radialis in 60 normal adults, but much lower in the 60 infants [19]. However, whether any deviation resulted from variations due to different measurers remains unclear. A survey of these age groups after 11 years old is necessary to determine when the PPT values approximate adult levels.

A child may have already developed an A-TrP at the origin of the brachioradialis muscle at 4 years old, although the exact timing of the development of the A-TrP is unclear. For each age group of children, the A-TrP of the brachioradialis muscle is more irritable than the corresponding MTrP. The tendon attachment of a muscle may likely be still immature (not strong enough) during childhood so that it suffers injuries easily. However, analysis of the gender differences reveal no significant differences between the genders in terms of the mean pain thresholds at the three measured sites even though boys are generally more active than girls in Oriental culture with respect to daily activities. In a similar study by Kao et al on an adult population, the MTrP of the brachioradialis muscle was determined to be more irritable than the A-TrP [19]. The timing of this change in muscle irritability remains unclear. Further studies are required to clarify these questions.

Dommerholt thoughtfully discussed the issue regarding the concept of A-TrP [30]. Simons proposed the concept of the A-TrP to explain pain at the muscle-tendon junction in people with MTrPs, based on the assumption that taut bands would generate sufficient sustained force to create localized enthesopathies. However, to date, no convincing evidence has proven that the tension generated in shortened sarcomeres in a muscle belly could generate passive or resting force throughout the entire taut band [30]. On the contrary, the force generated by individual motor units is always transmitted laterally to the muscle's connective tissue matrix [31,32]. Dommerholt also argues that there is considerable evidence to suggest that the assumption that muscle fibers pass from tendon to tendon is without basis, concluding that the development of the so-called "attachment trigger points" as a result of increased tension by contracted sarcomeres in MTrPs is not clear, and more research is needed to explain the clinical observation that MTrPs appear to be linked to pain at the muscle-tendon junction [30]. However, the term "A-TrP" is still used even if the mechanism of hyperirritability is unclear because the site is the tendon "attachment" region and it is definitely a hyperirritable spot.

Based on clinical and basic studies on MTrPs, the pathophysiology of MTrP has been much clarified [1,3,7,21,33-36]. Hong and Simons hypothesized that there are multiple MTrP loci in an MTrP region [7]. The sensory component of the MTrP locus is the sensitive locus or local twitch response (LTR) locus [2] at which pain, referred pain, and local twitch responses can be elicited. Meanwhile, the motor component is the "active locus" [37], from which spontaneous electrical activity(SEA)(mainly endplate noise [EPN]) can be electromyographically recorded. This was later defined as the "SEA locus" [7] or "EPN locus" [38,39]. An LTR locus is a sensitized nociceptor (free nerve ending) [27,40] and an EPN locus is a dysfunctional endplate with excessive release of acetylcholine (ACh) quanta [33-35,41,42]. An SEA locus is in close proximity to an LTR locus, and both interact to form the taut band and to facilitate MTrP formation [1,33]. Excessive leakage of ACh molecules (not simultaneously, so that EPN can be recorded) can cause the focal contraction of sarcomeres in the endplate zone to form a contraction knot as a taut band, which has been demonstrated in several morphological studies [43-48]. Impaired circulation and increased energy consumption in the contraction knot (trigger region) can cause a vicious "energy crisis" cycle [49]. This phenomenon in the MTrP has been further supported by recent biochemical studies that demonstrated a high concentration of inflammation or pain related substances in active MTrP regions [50,51]. Based on the above findings, Simons suggested an "integrated hypothesis for MTrP," that considers excessive ACh release, sarcomere shortening, and the release of sensitizing substances as the essential features of MTrPs [1,35,36]. Tissue ischemia and hypoxia in the contraction knot may induce the secretion of sensitizing substances that cause pain. The sensitizing substances can further cause abnormal ACh release, which activates a vicious cycle. These three essential features relate to one another in a positive feedback cycle that is self-perpetuating once started [1,34-36]. With effective MTrP therapy, this vicious cycle can be interrupted at several points in the cycle. However, researchers are still uncertain whether an "abnormal ACh release" initially occurs to sensitize the nociceptors via peripheral sensitization or whether an "inflammatory reaction" initially causes the release of inflammatory and pain substances, and then induces abnormal ACh release. In fact, the findings by Shah [50,51] support either hypothesis because the inflammation reaction can also be elicited by the muscle ischemia in the contracture knot.

Gunn considered the neuropathic lesion to be the primary mechanism of MTrP formation because EPN can be recorded in the MTrP region and facet joint lesions can activate MTrPs [52]. Hong agreed with this hypothesis for latent MTrP formation, but not for the process of latent MTrP activation [53]. The formation of a latent MTrP may be due to minor radiculopathy from minor repetitive stress to the spine while a baby is growing up. Minor radiculopathy may cause excessive ACh secretion at the neuromuscular junction, subsequently inducing electrotonic potentials (endplate noise) in the neuromuscular junction to cause the focal contraction of sarcomeres in the endplate zone (contracture knot).

Partanen et al suggested a different mechanism of MTrP formation, and concluded that MTrPs are related to painful muscle spindles in taut bands [54]. However, this hypothesis cannot explain the focal shortening of sarcomeres that occurs only in the endplate zone (contraction knot) [45,48].

Repetitive activity of a muscle may or may not cause MTrP formation. In a previous study, the PPT of common finger extensors can be reduced after piano practice, i.e., with repetitive finger activity [16]. A recent study demonstrated that an MTrP (a sensitive spot in a palpable taut band with reduced pressure pain threshold) of the extensor digitorum muscle could be induced by the repeated eccentric exercise of that muscle [55]. Unfortunately, in our study, no correlation was observed between the activity level of the students and their mean PPT values, neither when the differences in PPT of MTrP between dominant side and non-dominant side nor when the differences between "active" and "normal" children were compared. In the study by Kao [19], the PPT of MTrP between different sides had no significant difference. Although the dominant upper limb conducts more activity than the non-dominant one, the majority of daily activity requires the involvement of both sides.

Regarding the activity levels of children in the current study, the extra activity appears to be inadequate to change the irritability of an MTrP in the brachioradialis muscle. This is probably due to the very active lifestyle of children. Another possibility is that different types of sports may provide different levels of influence. We attempted to compare the difference in PPT of MTrP for children involved in different types of sports, but no obvious correlations were found. Further studies on different muscles across different age groups are required to clarify these questions.

The brachioradialis muscle was selected for this study because the PPT measurement of this muscle is relatively easy. In fact, many other muscles contract more frequently than the brachioradialis in daily living activities.

Only age groups from 4 to 11 years were involved in this study. The sample size for the 4 years old age group was smaller than those of the other groups because children below 5 years old are usually unwilling to participate in this type of study and are also less cooperative. Initially, we attempted to approach 12-year-old children, but for some unknown reasons, recruiting such children was difficult. One possible reason is that they are in their last year at elementary school and are involved in academic examinations, and another is that they are just beginning to be teenagers and therefore lack interest.

Another problem in this study is the reliability of the assessment of pain threshold in children. They might respond to pressure compression inconsistently. Consequently, the standard deviation of PPT is relatively large.

Further studies that include all possible age groups, larger sample sizes, and the use of a reliable measuring tool are necessary to conduct a complete survey.

## Conclusions

In summary, in the age group 4-11 years, the bony attachment region of the brachioradialis muscle is more irritable than the mid-belly and both of these regions are more irritable than the muscle/tendon junction site of the same muscle. All three regions are much more irritable in children 3-4 years old, less so for 6-year-olds, and least for those 7-11 years old. A child may develop A-TrPs and latent MTrPs in the brachioradialis muscle at 4 years old. The pain threshold increases gradually with age until 9 years old.

## Abbreviations

MTrPs: myofascial trigger points; A-TrPs: attachment trigger points; PPT: pressure pain threshold; ANOVA: analysis of variance; LTR: local twitch response; EPN: endplate noise; SEA: spontaneous electrical activity; Ach: acetylcholine.

## Competing interests

The authors declare that the paper have no competing interests.

## Authors' contributions

All authors participated in critical revision and approved the final manuscript. TIH, MJK and CZH conceived the study and supervised its design, execution and analysis and participated in the drafting and critical review of the manuscript. FCK and YLH did data management and statistical analyses. CZH and LWC wrote the paper with input from all investigators.

## Pre-publication history

The pre-publication history for this paper can be accessed here:

http://www.biomedcentral.com/1471-2474/13/13/prepub
